# Intravenous Injections of Nuclein in Tuberculosis

**Published:** 1910-07

**Authors:** 


### Intravenous Injections of Nuclein in Tuberculosis.

Edgar P. Ward presents a new theory as to the action of nuclein in tuberculosis, in which disease he administers the remedy in a novel way. In every case of tuberculosis which he has examined within the past two years he has found a decrease in the specific gravity of the blood with a corresponding diminution of the percentage of hemoglobin. There is also a lessened number of red blood corpuscles, with an increase of the deformed cells, or poikilocytes, as they are called. These changes are proportionate to the severity of the disease. As a consequence of the low percentage of hemoglobin there is a marked deficiency in the oxygen-carrying power of the blood.

This blood condition Dr. Ward has found to yield rapidly under the influence of intravenous infusions of a solution of sodium tritico-nucleinate, standardized to one milligram of organic phosphorus to each cubic centimeter. A physiologic salt solution is used as a vehicle.

Dr. Ward believes that the value of nuclein depends not so much upon its property of producing leukocytosis as upon its power of restoring the percentage of hemoglobin and of the number of red cells, while at the same time reducing the number of poikilocytes and increasing the specific gravity. In other words, the nuclein acts as a "blood-builder." That all these changes take place when the nuclein-saline solution is administered is shown by the author's detailed report of fifteen cases.

The nuclein-saline solution is given intravenously, under strict aspetic conditions. The dose of the nuclein solution used varied from thirty to sixty minims. This is diluted with a salt solution consisting of calcium chloride, 0.25; potassium chloride, 0.10; sodium chloride, 9.00; water, 1000. One ounce of the mixture is allowed for each twenty pounds of the patient's weight, eight ounces of the combined nuclein solution being an average dose for a person of ordinary weight. The solution is introduced into the median cephalic or median basilic vein, about twenty minutes being required for the completion of the transfusion.

In practically every case thus treated there has been a rapid increase in the percentage of hemoglobin, while the number of erythrocytes and the percentage of poikilocytes was diminished. In the majority of cases there has been a corresponding improvement in the patient's condition, shown by fall of temperature, slowing of the pulse and increase of weight. In forty-eight cases thus treated, practically all advanced, Ward has had but five deaths. Nine patients out of the fifteen cases reported in detail recovered, four were improved and two died. These results are certainly unusual.

Dr. Ward's deductions are as follows:

1. If there is not a net increase in the percentage of hemoglobin in two weeks' time, we do not expect any permanent result.

2. Even where there is no net increase in the hemoglobin, the treatment aids in keeping these people upon their feet.

3. With a constant increase in the percentage of hemoglobin, when the same has reached 85 to 100 per cent, and remains at this point for one month after all treatment has ceased, the patient may be declared well.—*Medical Record.*

---

"Science knows that the evils of intemperance are due to the nature of the drink rather than to the weakness of the drinker."

"This curse of alcoholism with its open saloon, its crowded graveyards, and its mothers in tears, is out of harmony with the twentieth century and it must go."

"Law is embodied sentiment. Sentiment against alcohol must first be drilled into the convictions and habits of the people before it manifests itself in the ballot box."

"The cause of temperance will ever be a fluctuating reform until it is based on the education of the people as to the proven facts about the nature and consequent effects of alcoholic drinks upon the human system. When these facts become a part of public knowledge, dating from childhood, the saloon problem will be solved."—MARY HANCHETT HUNT.

